# Clinical Characteristics and Management of Angioedema Attacks in Polish Adult Patients with Hereditary Angioedema Due to C1-Inhibitor Deficiency

**DOI:** 10.3390/jcm10235609

**Published:** 2021-11-29

**Authors:** Katarzyna Piotrowicz-Wójcik, Małgorzata Bulanda, Aldona Juchacz, Joanna Jamróz-Brzeska, Jacek Gocki, Krzysztof Kuziemski, Robert Pawłowicz, Grzegorz Porebski

**Affiliations:** 1 Department of Clinical and Environmental Allergology, Jagiellonian University Medical College, Botaniczna 3, 31-503 Krakow, Poland; katarzyna.piotrowicz-wojcik@uj.edu.pl (K.P.-W.); gosia.lesniak@uj.edu.pl (M.B.); 2 Greater Poland Center of Pulmonology and Thoracic Surgery, Szamarzewskiego 62, 60-569 Poznan, Poland; juchacz.aldona@gmail.com; 3 Department of Immunology and Allergy, Medical University of Lodz, Tadeusza Kościuszki 4, 90-419 Lodz, Poland; jamrozjoanna1@gmail.com; 4 Specialist Clinical Practice Allergoderm, Jagiellońska 111/3, 85-027 Bydgoszcz, Poland; jacekgocki@poczta.onet.pl; 5 Department of Pulmonology and Allergology, Faculty of Medicine, Medical University of Gdansk, Smoluchowskiego 17, 80-214 Gdansk, Poland; k.kuziemski@gumed.edu.pl; 6 Department and Clinic of Internal Medicine Pneumology and Allergology, Wroclaw Medical University, Marii Skłodowskiej-Curie 66, 50-369 Wrocław, Poland; robert.pawlowicz@umed.wroc.pl

**Keywords:** hereditary angioedema, C1-inhibitor deficiency, bradykinin-mediated angioedema, treatment, emergency

## Abstract

Hereditary angioedema (HAE) due to C1-inhibitor (C1-INH) deficiency is a rare disease characterized by recurrent swellings. This study aims to determine (i) the clinical characteristics of the HAE patient population from Poland, and (ii) real-life patients’ treatment practices. A cross-sectional study involved 138 adult HAE patients (88 females, 50 males) treated in six regional HAE centers in Poland. Consecutive patients during routine follow-up visits underwent a structured medical interview on the clinical characteristics of the course and treatment of HAE attacks within the last six months. A total of 118 of 138 patients was symptomatic. They reported in total 2835 HAE attacks predominantly peripheral and abdominal, treated with plasma-derived C1-INH (61.4%), icatibant (36.7%) and recombinant C1-INH (1.9%). An amount of 116 patients carried the rescue medication with them while traveling, and 74 patients self-administrated on demand treatment. There were twice as many symptomatic women (*n* = 78) as there were men (*n* = 40). Women treated their HAE attacks significantly more often than men. Older patients (≥65 years) reported a longer delay in diagnosis, and practiced the self-administration of rescue medication less frequently in comparison to other patients. Clinical features of the surveyed population are similar to other European, but not Asian, HAE patient groups. Self-administration still remains an unmet medical need. Some distinct HAE patients may require special attention due to the severe course of the disease (females) or a delay in diagnosis (the elderly).

## 1. Introduction

Hereditary angioedema due to C1-inhibitor deficiency (HAE) is a rare, autosomal dominant disease. It is characterized by recurrent, unpredictable episodes of angioedema [1]. Swelling episodes affect the skin, mucous membranes, e.g., the mouth, gastrointestinal tract, or larynx [2]. Increased vascular permeability leads to excessive fluid displacement and severe angioedema attacks that could be spontaneous or triggered by some factors such as trauma, psychological stress, pressure, infection and many others [3]. In our study, we aim (i) to determine clinical characteristics and (ii) current practice in the management of acute HAE attacks in Polish adult patients with HAE in comparison to cohorts in other European countries (Switzerland [4], the UK [5], Italy [6], Greece [7], Sweden [8], Serbia [9], Slovenia [10], Romania [11], Belarus [12] and Austria [13]) and outside of Europe (Asia [14,15,16], the United States of America [17,18,19,20] and Canada [21]). Nationwide cohort studies were performed in many countries to assess the clinical profile of patients, evaluate their treatment and identify factors influencing the course of the disease, but most likely, recently, there has been no cohort study involving such a large group of HAE patients from countries located in Central and Eastern Europe with a focus on the above-mentioned research goals.

## 2. Materials and Methods

### 2.1. Study Sample, Setting and Design

**Figure 1:**
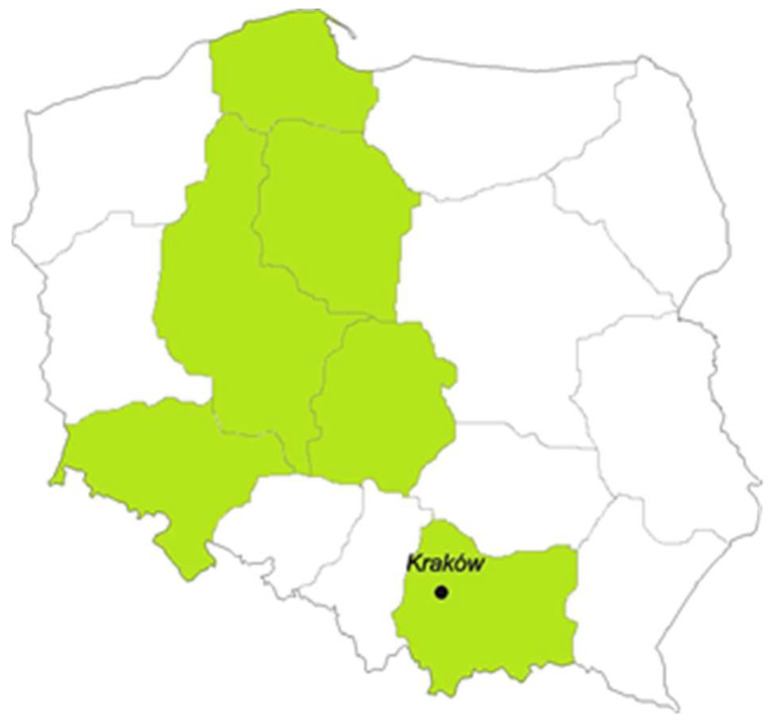
Regions involved in the study (projected on a map of Poland).

Patients from HAE centers located across Poland were enrolled into the study (Figure 1). Six centers accepted an invitation to participate in the survey: Krakow, Łódź, Toruń, Gdańsk, Wrocław and Poznań. All patients fulfilling the diagnostic criteria for HAE according to the current guidelines [2,22] were qualified for inclusion. We excluded from the study all patients under 18 years old and pregnant women due to ethical reasons and because pregnancy may strongly affect a clinical course of the disease [23]; thus, this could have impacted the representativeness of the results. Patients ≥ 65 years of age were assigned to the “elderly” group according to the cut-off point used in the work of Bygum et al. [24] and in accordance with the recommendations of the European Union and the WHO [25]. The other patients formed the younger group.

### 2.2. Data Collection

The survey was conducted between January 2018 and June 2019. Doctors in individual centers participating in the study undertook a structured medical interview among all patients with HAE that arranged a routine follow-up visit (both symptomatic and asymptomatic). If it happened in individual cases that a patient from a given center did not show up for a visit within scheduled time frame, the doctor contacted them by phone to conduct an appropriate investigation. As a result, all patients with HAE who were under the constant care of a given doctor in the center participated in the study. The issues included in the interview involved baseline personal and anthropometric data, followed by clinical data on disease course, symptoms and signs, as well as medication use in the last 6 months. This recall period was selected based on previous publication employing it [26]. Term “long-term prophylaxis”, used later in the text, refers to regular use of danazol or tranexamic acid aimed at preventing HAE attacks. Term “short-term prophylaxis” refers to treatment of patients with on-demand medications for HAE attacks to minimize the risk of swelling associated with situations where this risk is increased (e.g. dental procedures).

### 2.3. Statistical Analysis

Descriptive analysis was performed using median and percentage distribution of the population of enrolled patients, as appropriate. Two-tailed z-test to compare single proportion to population estimate, two-tailed z-test to compare two proportions and two-sample t-test of unequal variances and Mann-Whitney U tests were preformed to evaluate the data. Statistical analysis was performed by Julia version 1.6.2 standard library (open source (https://github.com/JuliaLang/julia), MIT License; accessed on 10 September 2021)).

## 3. Results

### 3.1. General Characteristics of the Study Group

We collected data from 138 adult patients (88 females and 50 males) at the mean age of 42.3 years (range 18–77, SD 15.5, median 39) (Table 1). The mean age at the onset of symptoms was 12.7 years (range 1–70, SD 8.7, median 11 years). Patients below this threshold in the studied period had significantly more attacks (mean 28.1) in comparison to patients who developed their first HAE symptoms later (mean 12.6 attacks), *p* < 0.0001. The mean delay in diagnosis was 15.2 years (range 0–56, SD 13.7, median 11 years). Three patients from HAE families were diagnosed before they developed symptoms. A total of 124 patients had HAE type 1 (90%) and 14 patients—HAE type 2 (10%). A positive family history was present in 120 patients (87.6%). The family history of HAE-related deaths, unnecessary surgeries and intubations due to laryngeal attacks were reported in 33.8%, 16.3% and 7.4% of the patients, respectively.

**Figure 2:**
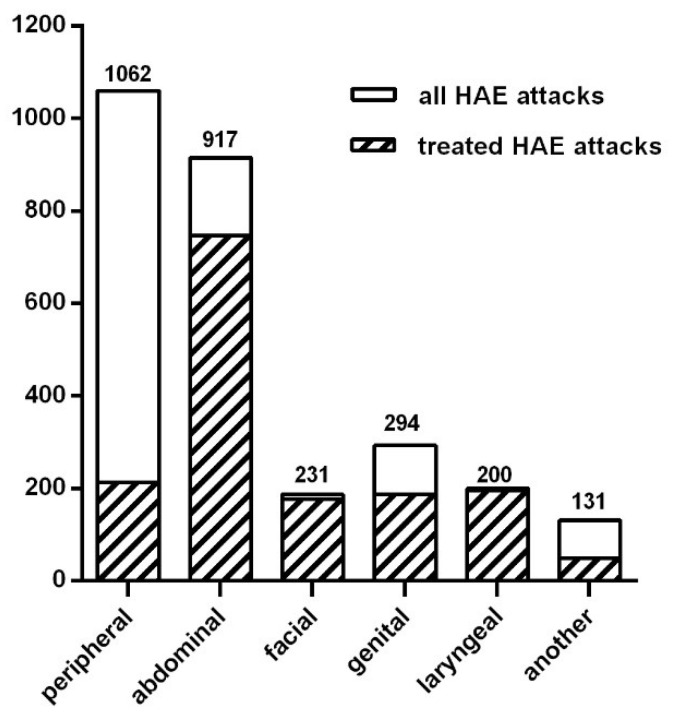
Numbers and localizations of HAE attacks during last 6 months.

In total, 118 of 138 patients were symptomatic within the previous 6 months. They reported in total 2835 HAE attacks. In this study, we defined an attack as the occurrence of swelling in one location at a given time point. In most occasions, particular attacks affected a single site at a single time point, and in 31.6% occasions two or more attacks occurred at the same time. Localizations of attacks are presented in Figure 2. The most common symptoms were (i) peripheral swellings occurring in 85% of symptomatic patients (median: 5, mean: 7.9, range: 0–48 attacks per 6 months per patient) and (ii) abdominal attacks occurring in 84% of symptomatic patients (median: 4, mean: 6.8, range: 0–30 attacks/6 months per patient). Laryngeal attacks were reported in 31% of patients within 6 months preceding the survey. The numbers of patients with high (≥1 attacks/week), moderate (<1/week and ≥1 attacks /month) and low (<1/month and ≥1 attacks /six months) frequency are presented in Table 2. In the group of patients with the highest rate of attacks, the frequency (≥1 attacks/week) mean number of attacks in the studied period was 41.6 (range from 21 to 110). The main triggers of HAE attacks reported by patients were stress (85 patients), physical trauma (82 patients), exertion (57 patients), infections (55 patients), and also food (18 patients). The foods mentioned by the patients included chocolate, crisps, grapes, onion, meat, pickled cucumbers, protein supplements, and strawberries. In total, 63.6% of patients complained of prodromal symptoms that preceded attacks: erythema marginatum (30 patients), weakness (20 patients), a skin sensation (16 patients), anxiety (14 patients) or a feeling of pressure in the abdomen (8 patients).

A total of 103 of 118 symptomatic patients took an on-demand treatment for HAE attacks in the last 6 months. Plasma-derived C1-INH (pdC1-INH), icatibant and recombinant human C1-INH (rhC1-INH) were used in 81, 55 and 10 patients, respectively. Most of the laryngeal, abdominal, facial and genital attacks were treated with the above on-demand medicines: 98%, 81.5%, 77% and 63.5%, respectively (Figure 2). Additionally, 20.1% of peripheral attacks were treated with disease-specific drugs. Taking into account the particular drugs, the percentage of HAE attacks treated with pdC1-INH was 61,4%, with icatibant 36,7% and with rhC1-INH 1,9% (Table 2). An amount of 28 patients reported the need for an additional dose of the emergency drug for an HAE attack during the suited period (8, 17 and 3 patients for pdC1-INH, icatibant and rhC1-INH, respectively). For use in an emergency, 100 patients had a home storage of pdC1-INH: 71—icatibant; 15—rhC1-INH. In total, 74 patients had simultaneously more than one drug at home, while 6 patients did not have any on-demand drugs at home. Most of the patients (*n* = 116) carried the rescue medication with them while traveling. A total of 74 patients self-administrated an on-demand treatment, and 63 patients were seeking help in hospitals or outpatient’s clinics. The mean time of journey from home to a medical service point was 20 min.

In total, 68 of the studied patients received long-term prophylaxis (LTP) at any time in the past, 24 of them had used danazol only, 25 tranexamic acid only, and the other 19 had used both drugs, but at different times. Comparisons of patients on LTP ever in the past to the rest of the patients were performed for age, age at onset of symptoms, delay in diagnosis, frequency of attacks, sites of attacks (including peripheral, abdominal, facial, genital, and laryngeal localizations), as well as frequency of on-demand treated attacks. It was showed that patients under LTP ever in the past were slightly more often female (*p* = 0.028), had earlier onset of the disease (*p* = 0.001) and more laryngeal attacks in the last 6 months (*p* = 0.03). The other clinical features did not reach statistically significant differences, however the frequency of HAE attacks per patients per 6 months was as high as 23.4 in patients under LTP ever in the past in comparison to 16.1 in other patients. 23 of the studied patients used LTP within the last 6 months (16 individual used danazol, the other 7 tranexamic acid). The median frequency of HAE attacks in the last 6 months was 6 in this group, while in the remaining patients, it was 55, *p* = 0.064. Within the studied period, short-term prophylaxis (STP) was used by 31 patients for 55 occasions in total (42 times before dental procedures, the other indications included most commonly endoscopic examinations). Pd-C1INH was administer in all cases except four, in which patients used icatibant of their own accord, despite recommendations on preferred medication for short-term prophylaxis. Comparisons of patients who were given STP in the last 6 months to the rest of the patients were performed for age, age at onset of symptoms, delay in diagnosis, frequency of attacks, sites of attacks (including peripheral, abdominal, facial, genital, and laryngeal localizations), as well as frequency of on-demand treated attacks. It was showed that patients who were taking STP in the last 6 months had more facial HAE attacks (*p* = 0.001). The other clinical features did not reach statistically significant differences.

### 3.2. Comparison of Women versus Men

There were no significant differences between women and men in the mean age at the onset of symptoms: 12.8 years (range 1–29 years, median 10 years, SD 9.4) vs. 12.5 years (range 1–32 years, median 10 years, SD 7.6), respectively. Additionally, the mean delay in diagnosis was similar for females (14.7 years) and males (16.4 years) (*p* > 0.05), as well as the proportion of HAE type 1/type 2 (11.4% of women and 8% of men had HAE type 2) (*p* > 0.05). A positive family history of HAE was noted in 88% of female patients and 86% of male patients (*p* > 0.05).

The number of symptomatic female patients in the previous six months was twice as high as in the group of male patients. The details, including differences in the frequency of attacks in respect of sex, are shown in Table 2. During the last six months, both in women and in men, the peripheral and abdominal localizations of angioedema attacks were dominant (Table 3). Triggers inducing HAE attacks were reported by 92% female and 84% male patients.

Women treated their HAE attacks with on-demand drugs more often than men (Table 2). There were no statistically significant differences between female and male patients in on-demand treatments for HAE attacks in respect to the type of drug that was used (Table 2). A total of 97.6% of female patients and 85.7% of male patients had emergency drugs stored at home (*p* > 0.05). Self-administration was practiced by 81% of symptomatic women and 57.5% of symptomatic men (*p* = 0.007). Women having ever used LTP in the past did so a bit more often than men, but within the last 6 months, this difference was not statistically significant (Table 2). Additionally, in regard to STP, women used this treatment more often, but the difference in comparison to men was not significant, what could be attributed to a small number of patients in the group under STP within the studied period (*n* = 31).

### 3.3. Comparison of Older versus Younger Group

We identified 14 patients ≥ 65 years (the elderly), the other 124 individuals represented the younger group (Table 4). The delay in diagnosis was significantly longer in the elderly vs. the younger (26.5 vs. 14 years, *p* = 0.04). A positive family history of HAE was noted in 78.6% of elderly patients and in 88.4% of younger patients (*p* > 0.05). In the study group, there were more symptomatic patients in the younger patients (87.9%) than in the older ones (64.3%) (*p* = 0.0174). Differences in frequencies of HAE attacks between the symptomatic older and younger patients were not statistically significant (Table 4). There were no statistically significant differences between the elderly and younger patients in respect of the frequency of the usage of the on-demand treatment for HAE attacks and the type of drug that was used. Self-administration was practiced by 44% of the symptomatic elderly, and 83.7% of the symptomatic younger patients (*p* < 0.01). Additionally, the symptomatic younger patients more often had the emergency drugs stored at home in comparison to the symptomatic older patients (66.1% vs. 44.4%, *p* > 0.05).

## 4. Discussion

This was the first cross-sectional study on Polish HAE patients. The survey aimed to determine the clinical characteristics of patients from the perspective of current management practices in the treatment of acute HAE attacks, which is a rare clinical condition with a prevalence range from 1:60,000 to 1:100,000 in the general population [24,27]. We collected data from 138 adult patients (88 women and 50 men) from the network of regional centers and the main center coordinating the study in Krakow. It should be taken into account that the study did not include all Polish HAE patients, which was its limitation. However, taking into account the available data [28], our study group involved about half of the Polish HAE patient population. Patients came from randomly included centers, so they were not biased by any selection method. Taken together, our patient sample may be assumed to also be representative for a larger HAE patient population. Our data showed a higher prevalence of HAE type 1 (90% of the patients) over HAE type 2 (10%), confirming the findings from several other countries such as Switzerland (98.1%) [4], the UK (85%) [5], Italy (87%) [6], Greece (80.5%) [7], Sweden (93.2%) [8], Serbia (89%) [9], Romania (91.7%) [11], Belarus (84.4%) [12], Austria (80.2%) [13], Korea (90.8%) [16] and the USA (78.4%) [19]. The Slovenian cohort from 2013 consisted of five families with HAE type 1 (63%) and three families with HAE type 2 (37%) [10].

Our patients reported an onset of symptoms at a median age of 13 years (Table 1) with no statistically significant differences between patients with HAE type 1 and HAE type 2. Most patients experienced their first attacks in childhood or adolescence, and this early onset of symptoms may implicate a more severe clinical course [29]. In the Icatibant Outcome Survey [30] researchers found a wide difference in the age of HAE onset in the six countries compared (median: 13.0; median range: 9.5 (Italy) to 15.0 (France) years). At the time of the first symptoms, patients with HAE in Italy were significantly younger than patients with HAE in France, Germany–Austria and Spain [30]. The first symptoms developed later in Chinese and Korean HAE patients than in Caucasians [31], which may be associated with dissimilarities in the course of the disease in Asian patients [16]. Sex-related clinical characteristics of HAE patients were similar as in other HAE cohorts (Table S1). In our study, the number of symptomatic female patients in the previous six months was twice as high as in the group of male patients; also, the frequency of angioedema attacks was higher in the women group (Table 2). In other analyzed studies, women also dominated [4,6,12,13,14,16,19]. These observations could be explained by the participation of estrogens in triggering HAE symptoms and the greater willingness of women to undergo diagnostic tests. There were no significant differences between women and men in the mean age at the onset of symptoms.

As the number of data dedicated exclusively to older patients was limited, we also conducted a data analysis considering the division into groups of younger and older patients. In our analysis, in the elderly versus the younger group, the delay in diagnosis was significantly longer in the group of patients above 65 years of age. The mean delay in diagnosis was 26.5 years in older patients and 14 years in younger group (Table 4), which stayed in line with observations by Bygum et al. [24]. For comparison, the delay from other countries was: Switzerland (average 14 years, without family history) [4], the UK (HAE type 1—10 years; HAE type 2—18 years) [5], Greece (16.5 years) [7], Sweden (median 10 years) [8], Serbia (median 11 years) [9], Romania (15.6 years) [11], Belarus (19.3 years) [12], Korea (mean 7.8 years) [16] and the USA (8.4 years) [19]. It should be emphasized that HAE is still an underdiagnosed disease and the delay in diagnosis is too long. However, our findings may suggest improvements in HAE diagnosis over time in Poland. The diagnostic delay could be due to the rareness of the disease or to symptoms being mistaken for other more common diseases, such as histaminergic angioedema and acute abdominal illnesses, or even irritable bowel syndrome [8]. With the first symptoms of HAE, patients usually report to their family doctor; therefore, the education of health-care professionals is important. The early diagnosis of HAE is important to improve patients’ quality of life and even save their lives in the presence of life-threatening laryngeal or abdominal attacks. Additionally, in our study, the younger group showed symptoms slightly more often compared to the elderly (Table 4), but low numbers of the elderly group may have affected this result. The overview of the main clinical and demographic data and how they compare to similar cohort studies is shown in Table S1.

The clinical symptoms of HAE include attacks of slowly increasing self-limiting swelling of the subcutaneous tissue and/or mucous membranes. In our analysis 86% of patients were symptomatic during the preceding 6 months. Generally, most patients with HAE in Europe were symptomatic in the year preceding the survey: in Switzerland (75%) [4], Greece (≥98.7%) [7], Sweden (78%) [8], Serbia (90.9%) [9], Romania (95.2%) [11] and Belarus (87.5%) [12]. In Asians, the severity of HAE symptoms appeared to be milder than in Europeans [16]. The proportion of asymptomatic patients in Taiwan was reported to be higher than in Europe: 42.1% [32]. Patients often complain about prodromal symptoms that precede attacks. In Poland symptoms such as erythema, weakness, skin discomfort, anxiety or pressure in the abdomen before the HAE episode was reported by 63.6% of our patients. In other studies, prodromal symptoms were reported between 68% and 82.5% [4,8]. The main triggers that provoked HAE symptoms in our study were: stress, physical trauma, exertion, infections and also food. These observations corresponded to trigger factors, which were described by other authors [4,33].

The total number of HAE attacks within the last 6 months in both of our patient groups was 2835 episodes. The most common symptoms were peripheral swellings. Laryngeal attacks, being potentially life threatening, were reported in 31% of patients within 6 months before the survey. A relatively high percentage of reported laryngeal attacks may be attributed to the fact that patients might be not able to differentiate correctly the locations of swelling in the upper airway. Therefore, they might report symptoms in the larynx, pharynx and mouth together, what in turn might have resulted in the overestimation of the frequency of the laryngeal attacks in our study. However, Romanian patients also reported a quite high frequency of laryngeal symptoms (39.3%) during the preceding 12 months [11]. The other four observations were similar to the conclusions of studies from other European countries. For example, in Romania, the most frequent episodes involved peripheral (89.3%) and abdominal (77.4%) symptoms [11]. Additionally, in the UK, an analysis of the attack frequency showed that the most frequent were cutaneous swellings followed by abdominal swellings, with a considerable variation between individuals and centers [5]. In The Icatibant Outcome Survey, abdominal attacks predominated (57.8%), followed by attacks localized to the skin (41.7%) and larynx (6.6%) [30]. Different results have been reported in Asia. The incidence of gastrointestinal tract involvement was relatively minor in Korea, while the most frequently involved site in Korean HAE patients was the face (82.3%) [16]. Additionally, 55.1% patients in a Chinese study reported face edema [31]. No patient underwent tracheal intubation and there were no fatalities due to laryngeal edema over the 6 months of the observation period in Poland.

The choice of on-demand treatment in HAE attacks is affected by local availability. Almost all laryngeal and abdominal attacks were treated with emergency drugs (98% and 81.5%, respectively). In contrast, for instance, in the recent study from Belarus, it was reported that as many as 18 patients were treated with drugs known to be ineffective in HAE: antihistamines, corticosteroids, tranexamic acid and only nine patients were given adequate treatment (icatibant, i.v. C1-INH concentrate or infusion of fresh frozen plasma) [12]. Our patients most often chose to have pd-C1-INH and icatibant at home, and much less often rhC1-INH. Additionally, these drugs, in similar proportions, have been used to treat HAE attacks. The possible reason why rhC1-INH was used so rarely was precisely the significant limitations to its availability caused by the lack of a reimbursement during the study period. In a Swiss study [4], for acute HAE attacks, pd C1INH was administered to 56% of patients in the female group and 53% of the male group, and icatibant in 16% and 6%, respectively [4]. In Japan, the only drug available with reimbursement for acute HAE attacks in 2015 was plasma-derived C1-INH concentrate, whereas ecallantide and icatibant had not been approved for HAE treatment there [14]. The analysis of the database records in the USA (2006–2014) revealed that 68.8% patients reported C1-INH (i.v.) use and 62.8% reported using ecallantide and/or icatibant [18]. In turn, in a Canadian study, the most commonly used agents for acute attacks in patients with HAE were C1-INH (88.2%) and icatibant (79.4%) [21].

In our study, 63% of the symptomatic patients reported the self-administration of an on demand treatment. The younger symptomatic patients used a self-administration of emergency drugs at home significantly more often in comparison to the older patients (83.7% vs. 44%). Women more often than men treated HAE attacks and used self-administration, which could be attributed to the fact that female patients had a higher rate of attack frequency (Table 2). The percentage of older patients in Poland, same as patients in Greece, who are currently home treated could not be considered satisfactory [7]. There is a very strong need to perform trainings about self- or family administrations of on-demand drugs among the older group of patients. Self-treatment after careful training is the optimal HAE treatment that improves patients’ quality of life, especially in the area related to the unpredictability of edema episodes. Despite the fact that most patients had on-demand drugs at home, almost half of them sought help in the hospital during the study period. This may be due to the fact that, unlike patients, public hospitals in Poland do not have access to reimbursed drugs for HAE. Therefore, patients who do not self-administer medications at home come to the medical care facility for administration. Furthermore, patients are also advised to see a doctor even after the administration of the emergency treatment in the case of severe life-threatening attacks. Although the majority of patients (*n* = 116) adhered to the principle of carrying an emergency medicine with them when away from home, e.g., when traveling, a minority still disregarded this recommendation. Our patients in emergency cases were able to reach a medical care center relatively quickly, but the more time it took between their arrival and the administration of medications could vary considerably. Patients seeking help in their family doctors’ practices during the working day usually received it very quickly. However, in the case of patients going to emergency departments at hospitals, waiting times depended on triage [34], and the recognition of HAE attack as a serious condition requiring fast action may by insufficient [35] also in Poland.

In current practice in Poland, and introducing of LTP is a shared decision-making process based on the physician’s advice and the patient’s preferences, reflecting an individual approach to this treatment. At the moment, the only available drugs in Poland for LTP in HAE are off-label medicines—danazol and tranexamic acid. In our study, many patients never required LTP in the past (*n* = 68), but a limited number of patients (*n* = 23) used this treatment on a regular basis. This difference may be related to transient conditions exacerbating symptoms and prompting LTP (e.g., stressful situations), as well as with the discontinuation of danazol or tranexamic acid due to the presence of side effects and the lack of efficacy, respectively. The obtained results suggest that LTP was more willingly introduced to patients with frequent laryngeal attacks and an early onset of the disease likely related to more severe course of the disease [29]. STP was used mostly before dental procedures, what may suggest that patients taking STP suffered often from diseases requiring these procedures and triggering frequent facial attacks. On the other hand, frequent facial HAE attacks (independent from oral cavity diseases) might encourage to use STP more often before any dental procedures. When comparing patients with and without LTP in the past, the female gender slightly predominated, but a direct comparison between females and males showed only a borderline tendency in favor of females (*p* = 0.0458), which disappeared completely when the last 6 months were analyzed (Table 2). There were significant differences between females and males in regard to STP (Table 2). It seems, therefore, that both long-term and short-term prophylaxis did not significantly affect the differences in symptoms between females and males. The Icatibant Outcome Survey rates of long-term prophylaxis use varied among countries, ranging from 11.8% of patients in Germany to 55.4% of patients in the UK. Overall, the predominant prophylaxis medications used were androgens (56.4%) [30]. In Belarus, only 9 of 38 patients declared carrying on a prophylactic treatment with Danazol and 1 with tranexamic acid [12]. In turn, two-thirds of patients in the USA used prophylactic medications (primarily C1-INH products or androgens) on an ongoing basis over the past year [19], and despite this treatment, they still experienced some HAE attacks. In our data, patients reporting the usage of LTP within the last 6 months had a reasonably lower rate of HAE attacks compared to the remaining patients (median 6 vs. 55 attack per 6 months), but the difference was not statistically significant, which could be attributed to the fact that some patients used usually less-effective antifibrinolytics, and some of the others stopped danazol treatment within the studied 6 months period and, as a consequence, they had frequent HAE attacks again.

## 5. Conclusions

To sum up, our data provided evidence from a large population that HAE patients from Poland are similar in terms of basic clinical features to other already-described European populations, including subpopulations of female and older HAE patients, but not to the Asian HAE population. In addition, our data, derived from almost three thousands HAE attacks, on real-life local emergency treatment practices, provided further insight into the still unmet medical needs of HAE patients, namely, the insufficient proportion of HAE patients who self-administer an emergency treatment and who carried the rescue medication when being away from home. Finally, the outcomes of our survey clearly confirmed that some distinct HAE patient groups may require special attention due to a higher rate of attack frequency (female patients) or a delay in diagnosis (the elderly).

## Figures and Tables

**Table 1:** Demographics and disease characteristic of the study group.

Characteristic	The Study Group
HAE diagnosis, *n* (%)	
Type 1	124 (90)
Type 2	14 (10)
HAE in family history, *n* (%)	
Positive	120 (87.6)
Negative	18 (12.4)
Sex, *n* (%)	
Female	88 (63.8%)
Male	50 (36.2%)
Age, years	
Mean	42.3
Min, max	18, 77
Age at onset of symptoms, years	
Mean	13
Min, max	1, 32
Delay in diagnosis, years	
Mean	15.2
Min, max	0, 56

**Table 2:** Sex-related frequency of angioedema attacks.

Patient Groups and Clinical Features	Total	Women	Men	*p*
All patients	138	88	50	**0.0012**
Symptomatic patients (% of all patients in group)	118 85.5%	78 88.6%	40 80.0%	**0.0004** 0.1660
# angioedema attacks (Mean # attacks per patient)	2835 20.5	2097 23.8	738 14.7	**<0.001** **0.0214**
# treated angioedema attacks (Mean # treated attacks per patient) (with pdC1-INH, icatibant, rhC1-INH) *	1709 12.4 (1050, 627, 32)	1362 15.5 (848, 488, 26)	347 6.9 (202,139, 6)	**<0.001** **0.0143**
Patients with:				
≥1 attacks/week	45	34/78 (43.6%)	11/40 (27.5%)	0.0883
<1/week and ≥1 attacks/month	46	30/78 (38.5%)	16/40 (40%)	0.8744
<1/month and ≥1 attacks/six months	27	14/78 (17.9%)	13/40 (32.5%)	0.0738
LTP ever in the past (D, TA, D and TA)	68	49/88 (56%) (14, 19, 16)	19/50 (38%) (10, 6, 3)	**0.0458**
LTP in the last 6 months (D, TA)	23	14/88 (16%) (9, 5)	9/0 (18%) (7, 2)	0.751
STP in the last 6 months	31	23/88 (26%)	8/50 (16%)	0.181

The significant *p*-values are in bold. #, number; *, medications that were used first to treat a given HAE attack; D, danazol; LTP, long-term prophylaxis; STP, short-term prophylaxis; pdC1-INH, plasma-derived C1-inhibitor; rhC1-INH, recombinant human C1-inhibitor; TA, tranexamic acid.

**Table 3:** Sex-related localization of angioedema attacks during the last 6 months.

Clinical Features	Female Patients		Male Patients	
Localization	# of Patients (% of All Women)	# of AE	# of Patients (% of All Men)	# of AE
Peripheral	70 (80%)	774	31 (62%)	288
Abdomen	69 (78%)	648	31 (62%)	269
Face	39 (44%)	189	13 (26%)	42
Genital	32 (36%)	225	20 (40%)	69
Larynx	34 (39%)	167	8 (16%)	33
Another	24 (27%)	95	8 (16%)	36

AE, angioedema; #, number.

**Table 4:** Age-related clinical characteristic and frequency of angioedema attacks.

Patient Groups and Clinical Features	The Elderly (≥65 Years)	The Younger (<65 Years)	*p*
Patients, *n* (%)	14 (10.1)	124 (89.9)	
HAE Type 1/type 2, *n* (%)	14 (100)/ 0 (0)	110 (88.7)/ 14 (11.3)	0.1845
Sex Female/male, *n* (%)	10 (71.4)/ 4 (28.6)	78 (62.9)/ 46 (37.1)	0.5305
HAE family history (%)	78.6	87.9	0.3274
Mean delay in diagnosis (y)	26.5	14	0.04
Symptomatic patients, *n* (%)	9 (64.3)	109 (87.9)	0.0174
Patients with: ≥1 attacks/week	2/9 (22.2%)	43/106 (40.6%)	0.2776
<1/week and ≥1 attacks/month	3/9 (33.3%)	43/106 (40.6%)	0.6678
<1/month and ≥1 attacks/six months	4/9 (44.5%)	23/106 (21.7%)	0.1213

The significant *p*-values are in bold. y, year.

## Data Availability

The data and analysis scripts are available upon request from the corresponding author.

## Supplementary Materials

The following are available online at https://www.mdpi.com/article/10.3390/jcm10235609/s1, Table S1: Comparison of the study group to other groups in HAE cohort studies.
