# Supplementary material for: Clinical Characteristics and Management of Angioedema Attacks in Polish Adult Patients with Hereditary Angioedema Due to C1-Inhibitor Deficiency
Source: J Clin Med. 2021 Nov 29;10(23):5609. doi: 10.3390/jcm10235609 (PMC8658320; doi:10.3390/jcm10235609)
Supplement: Supplementary file 1 [file jcm-10-05609-s001.zip › jcm-1465270-supplementary.pdf]

Table S1. Comparison of to study group to other groups in HAE cohort studies

	2021 Guryanova (Belarus) [12]	2020 Banerji (USA) [19]	2019 Schoffl (Austria) [13]	2018 Jung (Korea) [16]	2016 Steiner (Switzerland) [4]	2016 Nordenfelt (Sweden) [8]	2015 Zanichelli (Italy) [6]	2015 Ohsawa* (Japan) [14]	2014 Psarros (Greece) [7]	**KPW et al. (Poland)**
methods	data from the medical records and questionnaire	web-based survey of patients with a self-reported physician diagnosis of HAE	email survey thorough doctors identified by patient organization, existing patient records	electronic medical records of patients with confirmed HAE were retrospectively reviewed	questionnaire supported by direct contact with the patient, the family physician, hospitals, or relatives	written questionnaire and then participate in a telephone interview	data collected from medical records	direct mail questionnaire to physicians experienced in HAE	completing the questionnaire by team members at the field visits to the patients	**completing the questionnaire by a doctor at a patient's regular visit**
inclusion	patients diagnosed with C1-INH-HAE at the Research Centre #	screening questions, adults	survey concerning HAE patients identified by MDs#	diagnosis of HAE	all patients fulfilling the diagnostic guidelines#	physician's diagnosis of HAE, including laboratory confirmation, adults	pts diagnosed in regional HAE centres#	pts diagnosed according to international criteria and local guidelines#	pts identified according to international consensus algorithm#	**physician's diagnosis of HAE, including laboratory confirmation, adults**
population	all patients from one Centre who completed questionnaire	patients identified and recruited through the US Hereditary Angioedema Association (HAEA)	patients reported by centres which were contacted to participated in the survey	patients diagnosed with HAE at 15 tertiary referral hospitals in Korea	all patients from selected regional centres	patients from national database who responded to the invitation to the survey	patients reported by regional HAE centres	patients reported by MDs from local network or MDs identified by authors through database search	patients from Greek HAE Registry	**patients from selected regional HAE centres**
number of patients	64	445 (who completed the survey)	137	65	104	102	983	171	116	**138**
female	60.9%	78.2%	56.2%	67.7%	54.8%	50%	53%	68.4%	47.4%	**63.8%**
patients age-mean/median	33.2/ 31.5	NR	NR	NR	44/NR	40/NR	NR/ 45	43.6/NR	43.4/ 38	**42,3/ 39**
patients with HAE family history	NR	78.4%	87.7%	58.7%	NR	87%	84%	76.6%	90.7%	**87.6%**
type I HAE	84.4%	78.4%	80.2 %	90.8%	98.1%	93.2%	87%	91.7%	80.5%	**90%**
onset of symptoms (y)	mean 12.3	mean 12.5	mean 12.6, median 6.5	mean 28.4	mean 11	median 12	NR	mean 24.2	mean 12, median 10	**mean 12.7, median 11**
diagnostic delay (y)	19	mean 8.4	median 15 mean 15	mean 7.8	mean 14	median 10	NR	mean 13.8	average 16.5	**mean 15.2, median 11**
symptomatic patients	87.5%	100% (inclusion criteria)	NR	NR	75% (attacks within 1 year)	78% (last year)	NR	70.1% (past year)	≥ 98.7%	**85.5% (last 6 months)**

bolded, data from the current study; * HAE with normal C1-INH levels in 3 patients; # adults and children; NR, not reported; m, month; y, years; MDs, medical doctors

Where necessary data in the table were recalculated on the basis of raw results provided in the papers.
